# Neural Networks for Time Perception and Working Memory

**DOI:** 10.3389/fnhum.2017.00083

**Published:** 2017-02-24

**Authors:** Sertaç Üstün, Emre H. Kale, Metehan Çiçek

**Affiliations:** ^1^Department of Physiology, Ankara University School of MedicineAnkara, Turkey; ^2^Brain Research Center, Ankara UniversityAnkara, Turkey

**Keywords:** attention, fMRI, frontoparietal network, time perception, working memory

## Abstract

Time is an important concept which determines most human behaviors, however questions remain about how time is perceived and which areas of the brain are responsible for time perception. The aim of this study was to evaluate the relationship between time perception and working memory in healthy adults. Functional magnetic resonance imaging (fMRI) was used during the application of a visual paradigm. In all of the conditions, the participants were presented with a moving black rectangle on a gray screen. The rectangle was obstructed by a black bar for a time period and then reappeared again. During different conditions, participants (*n* = 15, eight male) responded according to the instructions they were given, including details about time and the working memory or dual task requirements. The results showed activations in right dorsolateral prefrontal and right intraparietal cortical networks, together with the anterior cingulate cortex (ACC), anterior insula and basal ganglia (BG) during time perception. On the other hand, working memory engaged the left prefrontal cortex, ACC, left superior parietal cortex, BG and cerebellum activity. Both time perception and working memory were related to a strong peristriate cortical activity. On the other hand, the interaction of time and memory showed activity in the intraparietal sulcus (IPS) and posterior cingulate cortex (PCC). These results support a distributed neural network based model for time perception and that the intraparietal and posterior cingulate areas might play a role in the interface of memory and timing.

## Introduction

Despite the importance of time perception in human life and behavior, the brain mechanisms related to this function are not yet clear (Nobre and O’Reilly, 2004; Lewis and Walsh, 2005; Burr and Morrone, 2006). Humans can perceive a broad spectrum of time, although there is no receptor specifically dedicated to its perception as is found in the visual, auditory or olfactory systems (Grondin, 2010; Coull et al., 2011).

There are some theoretical approaches about how we perceive time. The pacemaker accumulator model and its extension the attentional gate model suggest that there is an internal clock which generates regular pulses and an accumulator which keeps track of these pulses (Treisman, 1963; Gibbon et al., 1984; Zakay and Block, 1995). There are other models which do not suggest any internal clock components but a distributed representation of time in terms of neural-network states (Karmarkar and Buonomano, 2007) or neural circuits (like corticostriatal) responsible for timing (Coull et al., 2004).

Previous studies have reported that several brain areas are responsible for time perception. In light of Functional magnetic resonance imaging (fMRI) findings, the basal ganglia (BG) have been thought to play a central, content-free and supramodal role in time perception (Coull et al., 2011). Likewise, the cerebellum has been considered to play a significant role in this function. Besides the subcortical activations in the cerebellum and BG, wide-ranging cortical network activations have been shown during timing tasks. Bueti et al. (2008a) suggested that the parietal cortex may have a role in perceptual and motor timing, while the extrastriate cortex is responsible for the timing of visual stimulus and movements. Ferrandez et al. (2003) showed that a stimulus duration comparison task activated the BG, supplementary motor area (SMA), ventrolateral prefrontal cortex, inferior parietal cortex and temporal cortex. This study suggested that the BG and SMA are related to the time-keeping mechanisms, while the frontoparietal network might be related to the attention and memory processes required for time perception. In another fMRI study, differences between perception of long and short time durations were examined. The results showed that, compared to short time durations, long time durations caused higher activations in the anterior cingulate cortex (ACC), presupplementary motor area, right frontal gyrus, bilateral premotor cortex and also in BG. In this study, the correlation between time duration and anterior cingulate activation showed that the activity in this brain area was related to the processes involved in attention and decision-making functions (Pouthas et al., 2005).

There is currently a discussion about the role of the insular cortex in time perception. Craig (2009a,b) has postulated a new time perception model, which attributes a central role to the anterior insular cortex (AIC). According to this model, the insular cortex forms a basis for the sense of the physiological condition of the entire body (introception) by collecting internal cues (such as the heart beat), whereby the related signals provide a basis for time perception (Craig, 2009a,b). It has been suggested that the physiological properties of our bodies change our perception of time and that this phenomenon is related to insular cortex activation (Wittmann et al., 2010).

All types of time perception tasks require working memory. To perceive the passage of time, we have to mark the beginning of the event by some means. As time passes, we have to update the information related to that marker. If a response is needed, we have to evaluate the initial point of time and the elapsed time. This presumably occurs with the aid of the working memory. Some behavioral studies have shown that the working memory affects time perception (Pan and Luo, 2012; Woehrle and Magliano, 2012), but there are no imaging studies that investigate the relationship between these two cognitive processes. A possible reason may be the difficulty of separating these two processes under the constraints of neuroimaging designs.

Our experimental design is called a foreperiod paradigm. In this paradigm a visual stimulus that moved across the screen was temporarily occluded and reappeared after the occlusion. The task involved making a perceptual judgement about occlusion time (Correa and Nobre, 2008). Moving stimuli have often been used in time perception paradigms because it is more similar to real world time perception experiences (O’Reilly et al., 2008). Besides simulating “everday life”, the foreperiod paradigm is easy to modify, which allows the creation of different task conditions. For these reasons, we recognized it as an appropriate paradigm to evaluate time perception and working memory.

The purpose was to reveal which distinct brain areas are related to time perception and working memory. We hypothesized that the working memory would induce activation in the prefrontal and parietal cortex, and that time perception would activate the prefrontal cortex, cerebellum and BG. We also predicted that the neural networks required for working memory and time perception would partially overlap.

## Materials and Methods

### Subjects

Fifteen healthy volunteers (8 male, aged 18–35 years, mean age = 22.46 ± 2.09) participated in the study. All volunteers were right-handed with normal or corrected-to-normal visual acuity and were naive as to the purpose of the experiment. All participants completed the Chapman and Chapman (1987) Handedness Inventory (its validity and reliability for use in the Turkish population was reported by Nalçaci et al., 2002). The Ankara University Clinical Research Ethical Committee approved this research project and written informed consent was obtained from all participants.

### Experimental Paradigm

**Figure 1:**
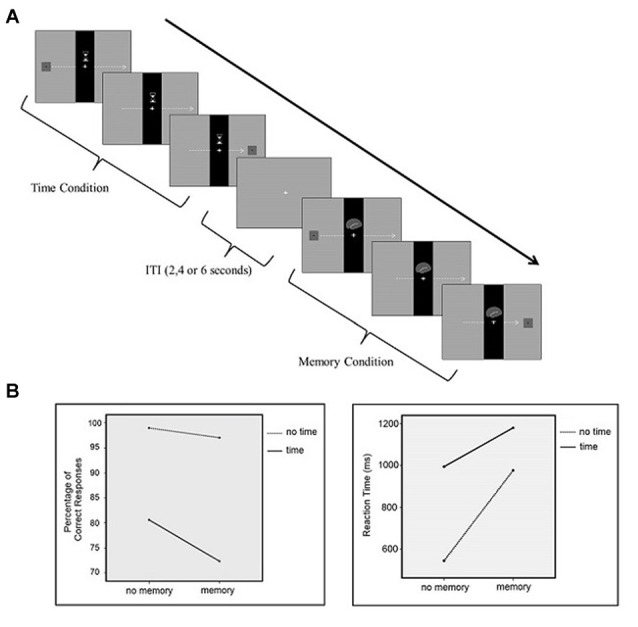
**(A)** Graphical representation of the sequence of events in each trial for all conditions. **(B)** Percentage of correct responses and reaction time (RT) results.

To examine our hypothesis, a visual paradigm was designed using Cogent2000 (Cogent2000 team at the FIL and the ICN, University College London, UK) which was run via MATLAB (Mathworks, Sherborn, MA, USA). The participants performed the tasks while undergoing fMRI which consisted of four different conditions: control, time perception, working memory, time-memory (dual). In all conditions, there was a black vertical bar in the middle of the screen with a gray background, which was constantly displayed during the trial. When present, the cue was displayed in the center of the bar/screen during the trial. Each condition had a unique cue associated with it, which was as follows: for the time perception condition, an hour glass; for the memory condition, a brain; for the time-memory condition, the former two cues combined; and for the control condition the bar alone. After presentation of the cue, a moving rectangle appeared from the left side of the screen and moved horizontally until it disappeared from the screen. The rectangle contained black dots and the number of the dots was a random integer from 1 to 4. When the rectangle reached the bar, the part of it that was under the bar was made “invisible” to the participant in order to induce the perception that the rectangle was passing under the bar. The initial speed of the rectangle and the speed when the rectangle was invisible were different. The speed either increased or decreased when under the bar, but it resumed its initial speed when the rectangle reappeared again. The number of dots also either increased or decreased from the initial dot count when the rectangle passed the bar. In the control condition, the participants were only required to press the button when the rectangle reappeared again on the right side of the screen. In the memory condition, the participants were asked to attend to the number of dots on the rectangle. If the number of dots increased by one, participants were required to press the right button of the fMRI response pad, and the left button if otherwise. In the time perception condition, the participants were asked to attend to the speed of the rectangle. They were instructed to press the right button if the speed of the rectangle increased while it passed under the black bar and press the left button if it decreased. In the time-memory condition, the participants were asked to attend to the speed of and the number of dots on the rectangle. They were asked to judge whether the number of dots increased by one or not. If not, they were asked to press the middle button. If the number of dots increased by one, they were to judge the change of speed which required pressing the right button if the speed of the rectangle increased and the left button if it decreased. Thus, in the time-memory condition the participants attended to both the speed of the rectangle and the number of dots simultaneously (Figure 1A). A fixation point was presented on a gray screen at intervals of 2000, 4000 or 6000 ms between trials.

The tasks were presented on a 28 cm × 37.5 cm screen with a distance of 72.5 cm from the participant’s eyes to the screen. The monitor resolution was 1024 × 768 pixels and the refresh rate was 60 Hz. The size of the black bar was 8.5 cm × 28 cm (6.70° × 21.85°) and the size of the moving rectangle was 4 cm × 3 cm (3.16° × 2.37°). The rectangle moved coherently from left to right with two possible speeds when it was visible to participant. The speeds were 5.26°/s and 6.99°/s. If the rectangle’s initial speed was 5.26°/s, its speed increased to 10.71°/s or decreased to 3.29°/s under the black bar and continued with this new speed until the rectangle exited the screen. If the rectangle’s initial speed was 6.99°/s, the speed increased to 12.45°/s or decreased to 5.03°/s under the black bar and continued with this new speed until the rectangle exited the screen. When the rectangles became visible on the right side of the screen, their speed changed back to their previous speed on the left side. One trial lasted 2500–6000 ms in total. The stimulus was visible for 2100 ms on average before it disappeared under the black bar and it remained invisible for 680 ms on average.

Instructions were given to participants before the fMRI scanning and during the fMRI acquisition participants did not speak. An event related fMRI design was used. There were four sessions (each lasting approximately 6 min) during the fMRI acquisition. Each session included 40 trials and consisted of 10 trials for each condition. The trials were rendered in a randomized order. The interstimulus intervals between trials were 2, 4 and 6 s arranged in a pseudo-randomized and logarithmic manner favoring shorter durations.

### Neuroimaging

Participants lay supine in the scanner with the response pad under their right hands. The visual stimuli were projected onto a projection screen situated behind the participant’s head and were visible via a mirror. Earplugs were used to muffle the scanner noise. A PC running Cogent 2000 via MATLAB controlled the presentation of the visual paradigm and recorded the participants’ responses.

A 3 Tesla Siemens Magnetom Trio MRI system was used for fMRI acquisition. The participants’ heads were immobilized with calipers built into the head-coil. High-resolution T1-weighted anatomical scans were obtained (Time to Repeat (TR): 2600, Time to Echo (TE): 3.02, Field of View (FOV): 256 mm, matrix: 256 × 256 and slice thickness: 1.00 mm). Functional scans were acquired in the axial plane using 46 3-mm slices with a 0-mm gap (TR: 2500, TE: 28, Matrix: 64 × 64, FOV: 192 mm, voxel size: 3 × 3 × 3 mm).

We obtained 136 TRs in each session. For all functional sessions, the first five images were excluded from the data for stabilization of the MR signal. There were four functional runs; as such, the data analyzed consisted of 524 images.

### Image Processing and Data Analysis

Analysis of the data was performed using SPM8 software (Wellcome Department of Cognitive Neurology, London, UK) run via MATLAB. The functional images were realigned to correct for movement artifacts. High-resolution anatomical T1 images were coregistered with the realigned functional images to enable anatomical localization of the activations. The structural and functional images were spatially normalized into a standardized anatomical framework using the default EPI template in SPM8, based on the Montreal Neurological Institute (MNI) averaged brain and approximating the normalized probabilistic spatial reference frame of Talairach and Tournoux (1988). Model estimation included a high-pass filter (256 s). Smoothing was performed with a 9-mm full-width half-maximum Gaussian kernel.

In the first level analysis, trials with incorrect responses were not included to eliminate the activations due to cognitive effort and task performance differences. Additionally, cognitive load was modeled with the reaction time (RT) so that RT modulated regressors were added for every trial. To eliminate the increased BOLD activation created by the presentation time, the stimulus duration was also included in the model. Duration and RT modulated regressors were orthogonalized w.r.t. to the unmodulated onsets (Mumford et al., 2015). We think we did our best to decrease the possibility of a limitation related to the performance data. We only analyzed trials with correct responses and modeled the effect of both stimulus duration and response times.

The neuroimaging data were statistically analyzed by a 2 (Time) × 2 (Memory) repeated measures analysis of variance (ANOVA) using a SPM8 flexible factorial design feature at the group level (Friston et al., 2007). The results were considered significant at *p* < 0.05 after FWE corrected for multiple comparisons. The main effect findings could be interpreted as giving time vs. control and memory vs. control contrasts. That’s why we performed direct comparisons between time perception and working memory conditions (t contrast) for assessing task related specific activations (again at the group level and *p* < 0.05, FWE corrected).

A region of interest (ROI) analysis was performed to further analyze the interaction effect. To this end, we obtained activated clusters for the interaction effect of time perception and working memory (see Table 1) via the MARSBAR toolbox of the SPM8 software and defined them as physiological ROIs (Brett et al., 2002). Using MARSBAR software, the mean percent signal change values for intraparietal sulcus (IPS) and posterior cingulate cortex (PCC) ROIs were calculated for each participant. We then used SPSS v.19 to analyze the percent signal change values by a 2 (Time) × 2 (Memory) repeated measures ANOVA.

**Table 1:** **Significant activations revealed by the 2 × 2 analysis of variance (ANOVA) analysis (*p* < 0.05 (FWE corrected))**.

			Talairach Coordinates		
Brain region	Cluster size	Laterality	*X*	*Y*	*Z*	*Z*-score
**Time condition**
Peristriate cortex	1090	L	−24	−80	−5	6.90
	599	R	23	−82	−2	6.45
Anterior cingulate cortex/	1121	Bilateral	5	30	35	6.48
Supplementary motor area
Insular cortex	170	L	−27	18	2	6.37
	287	R	28	20	0	6.20
Dorsolateral prefrontal cortex	768	R	46	11	21	6.12
Basal ganglia (globus pallidus)	175	L	−18	0	1	5.91
	220	R	11	3	3	5.50
Inferior parietal lobule	123	R	50	−39	41	5.25
Fusiform gyrus	11	L	−29	−62	−2	4.84
**Memory condition**
Peristriate cortex	1289	R	35	−82	0	6.51
Anterior cingulate cortex	200	L	−8	29	26	5.78
Basal ganglia (globus pallidus)	219	L	−10	0	1	5.60
Superior parietal lobule/precuneus	930	L	−26	−57	42	5.52
Fusiform gyrus	218	L	−42	−58	−5	5.42
Frontal eye field	35	L	−43	6	34	5.15
Talamus	36	L	−9	−27	−4	5.11
	33	R	7	−9	8	5.06
Cerebellum	27	R	12	−69	−14	5.07
Dorsolateral prefrontal cortex	23	L	−25	31	26	4.81
**Interaction**
Intraparietal sulcus	92	L	−42	−60	30	5.67
Posterior cingulate cortex	31	L	−7	−39	39	5.20
**Time > memory**
Intraparietal sulcus	13	R	52	−37	40	5.18
**Memory > time**
Superior frontal gyrus	91	L	−10	50	37	6.16
Posterior cingulate cortex	28	L	−5	−33	39	5.50
Calcarine sulcus	78	L	16	−55	11	5.66
	27	L	−7	−54	14	5.27

## Results

### Behavioral Results

We applied repeated measures ANOVA for the behavioral result analysis (separately for the percentage of correct responses and RTs) using SPSS v.19 software. The Bonferroni correction was applied for multiple comparisons.

The percentage of correct responses were analyzed using a 2 (Time/No Time) × 2 (Memory/No Memory) repeated measures ANOVA (Figure 1B left panel). The main effect of time was significant (*F* = 25.4; *p* < 0.0001). Participants’ percentage of correct responses in the time condition (80.3 ± 1.7) was lower than the control condition (98.8 ± 2.1). The main effect of memory was also significant (*F* = 24.6; *p* < 0.0001). In other words, participants’ percentage of correct responses in the memory condition (96.8 ± 2.0) was lower than the control condition (98.8 ± 2.1). The interaction between the time perception and working memory conditions was also significant (*F* = 7.5; *p* < 0.05). Performance was lowest in the time-memory condition (72.2 ± 1.8). Also percentage of correct response differences between time condition and working memory condition analyzed with paired *t*-test. Participants’ percentage of correct responses in the time condition was lower than in the memory condition and the difference was found significant (time > memory; *t* = −3.812, *p* < 0.05).

The RT results were analyzed using a 2 (Time/No Time) × 2 (Memory/No Memory) repeated measures ANOVA (Figure 1B right panel). There was a significant main effect of time (*F* = 75.6; *p* < 0.0001). Time perception RTs (993.4 ± 291.3) were significantly higher values than the control condition (543.4 ± 125.0). There was a significant main effect of memory (*F* = 123.7; *p* < 0.0001). The results from the memory task showed higher RTs (975.5 ± 140.1) compared to the control task (543.4 ± 125.0). Also, the time perception and working memory interaction was significant (*F* = 18.5; *p* < 0.05). The average RT for the time-memory condition was higher (1179.1 ± 174.7) than the control, time perception and working memory conditions. Moreover RT differences between time condition and working memory condition analyzed with paired *t*-test and the difference was not found significant (time > memory; *t* = 0.348, *p* > 0.05).

### Imaging Results

#### The Main Effect of Time Perception

**Figure 2:**
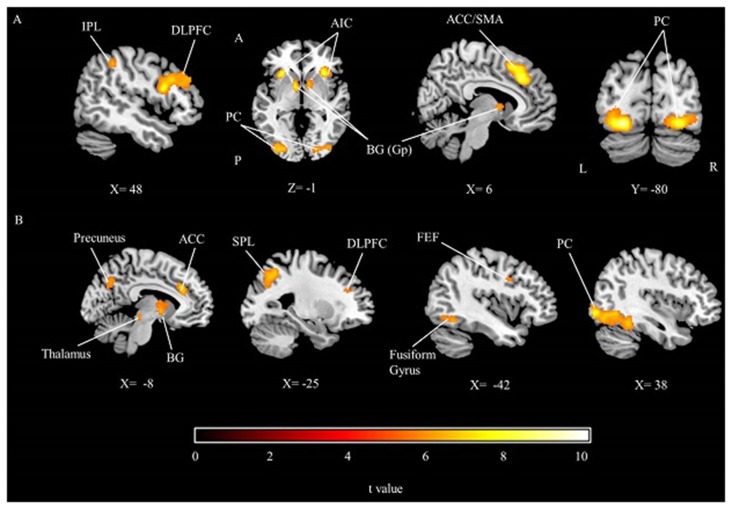
**The group results depicting significant activations related to (A)** the main effect of time perception,** (B)** the main effect of working memory, threshold at *p* < 0.05 (FWE corrected). IPL, Inferior parietal lobule; DLPFC, Dorsolateral prefrontal cortex; AIC, Anterior insular cortex; PC, Peristriate cortex; BG, Basal ganglia; Gp, Globus pallidus; ACC, Anterior cingulate cortex; SMA, Supplementary motor area; SPL, Superior parietal lobule; FEF, Frontal eye field; A, Anterior; P, Posterior; L, Left; R, Right.

The group results showed that while participants were performing the time perception task, the dorsolateral prefrontal cortex (DLPFC), inferior parietal cortex, insular cortex, middle prefrontal cortex, SMA (or ACC), frontal eye field, peristriate cortex and fusiform gyrus were significantly activated. In addition to the cortical activations, BG (Globus Pallidus) were also activated (Table 1; Figure 2A). Results show a right hemisphere lateralization. Most of the findings were more extensive in the right hemisphere, indeed the prefrontal and parietal cortex activity were significant only in the right side. Overall a distributed neural network with both cortical and subcortical components were significantly activated during the timing task compared to the control condition.

#### The Main Effect of Working Memory

During the working memory task, peristriate cortex, DLPFC, ACC, superior parietal lobe, precuneus, frontal eye field as well as the cerebellum, BG and thalamus were significantly activated (Table 1; Figure 2B). Memory task activated a left lateralized fronto-parietal network contrary to the timing condition which might be caused by the numerical nature of the memory task.

#### Interaction between Time Perception and Working Memory

**Figure 3:**
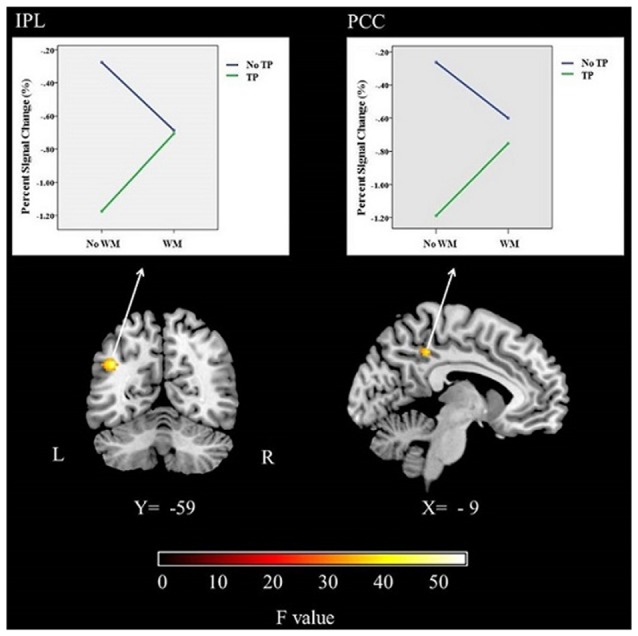
**The brain activations and related graphs of the interaction of time perception and working memory for the whole group (*p* < 0.05, FWE corrected).** IPS, Intraparietal Sulcus; PCC, Posterior cingulate cortex; L, Left; R, Right.

In this study, significant activations were found for the interaction between time perception and working memory. Activations were seen in the left IPS and left PCC (Table 1; Figure 3).

ANOVA was performed for percent signal change values obtained from left IPS and left PCC showed significant interaction effects (*F* = 42.46, *p* < 0.001; *F* = 76.63, *p* < 0.001, respectively; see Figure 3). The main effect of time was also significant for both ROIs (for IPS, *F* = 23.05, *p* < 0.001; for PCC, *F* = 25.05, *p* < 0.001). The main effect of memory was not significant (*p* > 0.05). The follow-up analysis showed that percent signal change was significantly different between timing condition and the dual (time-memory) condition and also the memory condition for both ROIs (*p* < 0.01). These results could suggest that while timing deactivates IPS and PCC, conversely the dual (time-memory), memory and control conditions activate these brain regions. However, the signal values might suggest a slight deactivation trend for the dual (time-memory) and also the memory condition for both regions, although the values were not significantly different from control condition (Figure 3). Activation of IPS and PCC during rest (the control condition) is in line with default mode network concept but the other findings need further discussion.

#### Specific Activations for Time Perception and Working Memory

**Figure 4:**
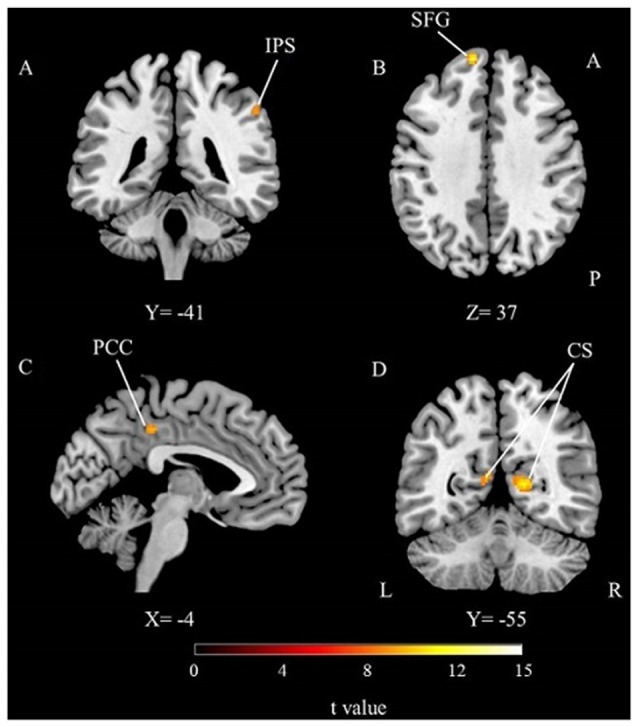
**The results of the direct comparison of time perception and working memory for the whole group (*p* < 0.05, FWE corrected). (A)** Activation from time condition > working memory condition comparison, **(B–D)** activations from working memory condition > time condition comparison, IPS, Intraparietal Sulcus; SFG, Superior Frontal Gyrus; PCC, Posterior cingulate cortex; CS, Calcarine Sulcus; A, Anterior; P, Posterior; L, Left; R, Right.

Direct comparison of timing with memory was performed to unravel task specific activations (Table 1; Figure 4). The time perception condition activated right IPS compared to the working memory condition. This suggests a right lateralized parietal engagement for timing mechanisms. On the other hand, working memory task activated the left superior frontal, left PCC and bilateral calcarine sulcus (primary visual cortex) regions. Frontal and cingulate cortical regions might be related to the working memory processes but calcarine activities might be more related to the engagement of primary visual processing of the stimuli.

## Discussion

We designed a visual paradigm which would help us show the brain mechanisms related to time perception and working memory processes. We found activations of fronto-parietal cortical network, together with the SMA, anterior insula and BG during time perception. On the other hand, working memory engaged a fronto-parietal and anterior cingulate cortical, as well as a BG and cerebellum activity. Both time perception and working memory showed a strong peristriate cortical activity. Last but not least, the results showed that memory and timing processes interacted in the IPS and PCC.

### Activations for Time Perception

Our findings revealed extensive right lateralized DLPFC and IPL activity during the time perception task. These results are in line with the results of previous lesion (Kagerer et al., 2002; Koch et al., 2002), transcranial magnetic stimulation (TMS; Jones et al., 2004; Alexander et al., 2005; Vallesi et al., 2007) and functional neuroimaging studies (Paus et al., 1998; Ferrandez et al., 2003; Pouthas et al., 2005; Bueti et al., 2008a,b) suggesting an especially important role of the right hemisphere, including the frontal and parietal cortices, in time perception. Kagerer et al. (2002) proposed that temporal processing of durations longer than 2–3 s requires an intact right hemisphere (mainly frontoparietal). It is claimed that while the DLPFC mediates the working memory aspects of the timing, the posterior parietal and anterior cingulate cortices are related to the attentional aspects of time perception (Lewiss and Miall, 2006).

The time perception condition lasted about 2.5–6 s in our paradigm and required participants to maintain features (shape and velocity) of the stimuli in mind, to predict the reappearance time after it disappeared under the black bar and to act by responding when it reappeared. The task likely requires global visuospatial attention processes to be engaged, which might also contribute to the more pronounced frontoparietal activity in the right hemisphere (Çiçek et al., 2007, 2009).

The time perception condition showed significantly more right IPS activity compared to the working memory task. This supports our view linking visuospatial attention processes with timing mechanisms in the IPS region. Significant right IPS activity was shown both for time vs. control and time vs. memory contrasts (coordinates were very close). So both the visuospatial timing task related (time > control) and specific timing related (time > memory) processes engaged IPS area. The brain processes for space, time and number magnitude are suggested to be interrelated (Dehaene and Brannon, 2011). Riemer et al. (2016) studied the effect of TMS application on the right IPS (in close proximity to the activated areas in the presented study) in terms of the performance parameters of a task differentiating space, time and number perception. Their results showed that right IPS engaged especially for the spatial and temporal aspects of the visual stimuli in line with our findings.

Bilateral extensive activations were shown in the SMA and ACC in the present study. O’Reilly et al. (2008), required healthy volunteers to perform a velocity judgement task similar to our paradigm and showed right lateralized prefrontal, SMA and premotor cortex activity. Ferrandez et al. (2003) suggested that the BG and SMA are related to the time-keeping mechanisms. In the study by Pouthas et al. (2005), which required participants to discriminate short and long intervals, they showed that the ACC and some motor cortical areas were activated during estimation of longer intervals. They suggested that the ACC is involved in attentional and decision-related aspects of timing. It is postulated that ACC activity is related to the updating of internal models (O’Reilly et al., 2013). The present study used a paradigm presumably requiring the manipulation of the internal models; the reappearance of the stimuli in an unexpected time (before or after the expected instant) should engage update processes of the brain. Thus, the SMA/ACC activity in our findings might be related to internal model update and/or decision-related aspects of time perception.

An interesting finding was bilateral activations in the AIC in the present study. The AIC has been suggested to contain introceptive (the sense of the physiological condition of entire body) representations, which might provide the basis for the subjective feelings and emotional awareness associated with the human body (Craig, 2009a,b). There are studies reporting that, the AIC is commonly activated with (and anatomically connected to) the ACC, suggesting that these areas constitute a neural network for the integration of introceptive stimuli (Craig et al., 2000; Stephan et al., 2003; Barrett et al., 2004; Critchley et al., 2004; Craig, 2009a). As Craig (2009a,b) postulated, one’s feeling of the physiological condition of the body processed mainly by the AIC could help construct the subjective present and pave the way for time perception. On the other hand, it was suggested that while the posterior insula encodes the passage of time, the anterior insula might be important for the reproduction of time intervals (Wittmann et al., 2010).

The BG were activated during the time perception condition. This finding is supported by TMS and lesion studies (Irvy and Keele, 1989; Nichelli et al., 1996; Malapani et al., 1998; Casini and Ivry, 1999; Koch et al., 2007; Lee et al., 2007). fMRI studies have shown activations in the BG (Paus et al., 1998; Ferrandez et al., 2003; Pouthas et al., 2005; Bueti et al., 2008b). Artieda et al. (1992) showed time perception deficits in patients with Parkinson’s disease, which is characterized by BG degeneration. Researchers suggest that the cerebellum and BG might be important for timing of shorter intervals, probably on a sub-second scale (Nichelli et al., 1996; Malapani et al., 1998; Casini and Ivry, 1999; Koch et al., 2008).

Activations for both time perception and working memory tasks showed that the peristriate cortex (extending in to the middle temporal gyrus (MT/V5)), was activated. The peristriate activity (Brodmann area 19) is likely more related to the secondary visual processing of our stimuli (Born and Bradley, 2005). On the other hand, it has been suggested that the MT/V5 region is involved in the temporal processing of visual motion (Bueti et al., 2008a). However, in our previous study, this region was also activated in relation to global spatial processing of visual stimuli (Çiçek et al., 2007). These findings might suggest that performing time perception and working memory tasks require participants to engage more attentional resources for the secondary processing of visual stimuli, especially the motion related aspects.

### Activations for Working Memory

In line with the previous visual working memory studies, the present study showed strong activation in the parietal (superior parietal lobule, precuneus) and frontal (DLPFC, FEF) lobes as well as in the peristriate cortex, fusiform gyrus, BG, cerebellum and thalamus during the working memory conditions (Goldman-Rakic, 1987; Smith and Jonides, 1998; Lewis et al., 2004; Osaka et al., 2004; Todd and Marois, 2004; Curtis, 2006; Marshuetz et al., 2006; McNab and Klingberg, 2007).

Interestingly the parietal and frontal activity was left lateralized in our findings. This could be due to the numerical nature of our working memory condition, which requires participants to keep the number of dots in mind and also to perform a simple calculation (subtracting one). Along the same line, left lateralized brain activity in frontal and parietal regions during the representation and processing of numerical quantities have been previously shown (Pinel et al., 2001; Pinel and Dehaene, 2010).

The direct comparison of memory vs. timing showed left superior frontal (or medial prefrontal), left PCC and bilateral visual cortex activity. These regions are popularly related to the default mode network which should be active during rest and deactivate during task performance. However recent reports suggest that these regions have some role in task related processes (Leech et al., 2012; Koshino et al., 2014; Oren et al., 2016). While medial prefrontal cortex was reported to be engaged during working memory, the PCC was suggested to modulate attentional load aspect of memory function (Oren et al., 2016). Koshino et al. (2014) showed activation of medial prefrontal and PCC during preparation of a working memory task. These findings suggest that our working memory condition engaged medial prefrontal and PCC areas which might be related to the task preparation and attentional load aspects of the paradigm. Bilateral primary visual cortex activation might be the result of high visual acuity needed for determining the dot numbers on the presented stimuli.

### Interactions of Time Perception and Working Memory Processes

Present study showed that memory and timing processes had an interaction in the IPS and PCC. ROI analysis showed that while timing deactivates IPS and PCC, the dual (time-memory), memory and control conditions activate these brain regions.

The time perception condition in our paradigm most likely required participants to perform spatial and temporal processing. On the other hand, the working memory condition engaged spatial working memory processes of brain. The two tasks were well matched at the level of visual stimulation. A moving black rectangle was presented in both conditions, in which it disappeared under a black bar and participants responded when it reappeared. In the time perception condition, participants attended to the velocity of the stimuli and judged its velocity change when passing under the bar. But in the working memory condition, they had to keep the number of dots in mind and manipulate the representation by performing a calculation. The time perception condition also probably engaged spatial working memory processes, but there was no temporal processing necessary for the working memory condition. Therefore, we suggest that while right IPS was activated during timing (as was proved by the presented study’s timing > memory contrast result), left IPS was deactivated in line with the right lateralized spatial and temporal processes required for our paradigm. Çiçek et al. (2007, 2009) reported mainly a right lateralized fronto-parietal activation during global spatial attention paradigms. On the other hand, working memory engaged IPS region, which might be the result of the numerical nature of our working memory paradigm (Menon et al., 2000).

Although default mode network and one of its main nodes, PCC, is activated during rest, this brain region was also reported to be engaged during some cognitive tasks (Spreng et al., 2009; Leech et al., 2012; Koshino et al., 2014; Oren et al., 2016). PCC was reported to be activated during an episodic retrieval task as opposed to the activation of precuneus (or dorsal part of PCC) during a working memory performance (Cabeza et al., 2002). PCC was suggested to gather continuous information from the world around us and also possibly from inside of our body automatically (Gusnard and Raichle, 2001).

PCC was suggested to be a connection hub between lateralized frontoparietal networks and subcortical brain regions (Leech et al., 2012). PCC was proposed to interact with other brain regions depending on the nature of the task demand. Leech et al. (2012) further suggested that PCC should be less active and less communicated with attentional systems during periods of a focused task. The timing task in our paradigm might be seen as a focused task resulting in the deactivation of PCC. On the other hand, working memory condition (apparently the dual condition as well) depends more on unfocused information gathering like the number of dot on the stimuli and awaiting for the response time which probably rather activated PCC compared to timing condition.

### Limitations

The main limitation of our study is a consequence of the nature of time perception processes. Attention and working memory are important cognitive components of time perception (Gu et al., 2015). Designing a time perception task which does not involve these components is quite difficult if not impossible. The time perception condition in this study requires the working memory because the participant must encode time information while the rectangle moved towards the bar (see “Materials and Methods” Section). Furthermore, the speed information must be maintained while the rectangle was occluded by the bar in order to determine whether the speed had increased or decreased (Harrington et al., 2010).

## Conclusion

The present study used a visual paradigm requiring timing judgements for the stimuli within the supra-second range. We propose that cognitively controlled timing processes engage a distributed brain network revolving around the right dorsolateral prefrontal and right intraparietal cortices as well as the AIC and BG (Lewiss and Miall, 2003; Teki, 2016). Introceptive signals processed mainly by the insula might construct the subjective present and add to the information supplied by the BG which might encode and maintain time intervals (Rao et al., 2001; Craig, 2009a,b). The insula and BG might contain a short-term memory buffer that receive processed input from a parietal network and transfer the information into the working memory in the prefrontal cortex (Rao et al., 2001; Kranczioch et al., 2005).

The PCC and intraparietal cortex might play a major role as an interface between episodic buffer aspect of working memory (Baddeley, 2000) and global attentional aspects of time perception. The PCC might play a major role as a connection hub between lateralized frontoparietal networks and subcortical brain regions like AIC and BG (Leech et al., 2012).

## Author Contributions

SÜ: substantial contributions to the conception and design of the work, acquisition, analysis and interpretation of data and writing the work. EHK: substantial contributions to the conception and design of the work, analysis and interpretation of data and revising the work critically. MÇ: project supervision, substantial contributions to the conception and design of the work, analysis and interpretation of the data, writing and revising the work critically. All authors: final approval of the version to be published and agreement to be accountable for all aspects of the work.

## Funding

This study was supported by the Ankara University Scientific Research Project Fund (project number, 13L3330004).

## Conflict of Interest Statement

The authors declare that the research was conducted in the absence of any commercial or financial relationships that could be construed as a potential conflict of interest.
